# Photon-counting spectral basis component material decomposition for musculoskeletal radiographs

**DOI:** 10.1038/s41598-020-70363-w

**Published:** 2020-08-17

**Authors:** Stefanie Beck, Thorsten Sellerer, Korbinian Mechlem, Jannis Bodden, Felix Meurer, Andreas Sauter, Julia Herzen, Franz Pfeiffer, Daniela Pfeiffer

**Affiliations:** 1 Department of Diagnostic and Interventional Radiology, Klinikum Rechts Der Isar, Technical University of Munich, 81675 Munich, Germany; 2 Chair of Biomedical Physics, Department of Physics and Munich School of Bioengineering, Technical University of Munich, 85748 Garching, Germany

**Keywords:** Bone, Trauma, Bone imaging, Radiography

## Abstract

As a very fast and non-invasive examination, conventional X-ray radiography is well established as the first line diagnostic imaging method of the human bone system. While major bone injuries such as fractures and dislocations are usually easily detectable on conventional X-ray images, more subtle injuries such as microfractures are often missed, leading to mistreatment and potential long-term consequences. The technology of Photon-Counting Dual-Energy Radiography (PCDER) yields the possibility to decompose conventional X-ray images into basis material images such as bone- and soft-tissue-equivalence images. The obtained basis material images offer significant advantages in terms of image contrast and image details over the raw attenuation image which shows an overlap of bone and soft tissue. Whereas the advantages of bone- and soft-tissue-equivalence images have been broadly discussed referring to bone subtraction images in the detection of pulmonary diseases, this method has not been considered for the analysis of musculoskeletal images until present. In this study we show that basis component equivalence images have high potential to improve the diagnostic accuracy of the detection of minor bone lesions during clinical trauma imaging. A reader study performed by three experienced radiologists compares the image quality of basis material images to a standard radiograph image of a non-fractured cadaveric hand.

## Introduction

Conventional X-ray radiography is the standard procedure for first line diagnosis of skeletal disorders. Although many injuries of the human bone system are missed during the initial radiological examination. The overall percentage of missed fractures in the extremities is estimated to be about 3.7%^1^. This problem particularly affects fractures of the hand and the wrist due to the high absolute numbers of hand and wrist fractures combined to the particularly high percentage of misdiagnosed fractures of wrist (4.1%) and hand (5.4%)^1–3^. The misdiagnosis often results in a delay of adequate therapy and thus the healing process and may cause further complications and long-term consequences such as non-union, wrist arthritis, avascular necrosis and disability^3,4^. It is also known that about 70% of the initially missed fractures are diagnosed in a second review^1^.

The reason for the failing diagnosis in the first examination underlies the subtlety of the injuries but also the limited spatial resolution and contrast properties of the available X-ray images. Conventional X-ray radiography shows an overall projection of the entire scanned area, with superposition of bone and tissue components yielding a limited image contrast and quality of the bone system. The superposition of bone and soft tissue in the conventional images is one of the reasons why many subtle bone injuries are not visualized properly on conventional X-ray images, while CT or MR reduce this superposition showing considerably better sensitivities for fracture detection^4–6^.

Spectral imaging offers a potential solution to this problem. Dual-energy radiography exploits the energy dependency of the attenuation caused by an object. Hence different materials can be discriminated by analysing the difference of the attenuation signals detected with different X-ray spectra^7–10^. This provides new prospects in medical imaging, since it allows material separation and provides quantitative information concerning the material composition.

Several clinical studies revealed a diagnostic benefit of material decomposition images in the field of dual-energy subtraction chest radiography, where bone- and tissue-selective images have resulted in an improved detection ratio of pulmonary nodules and masses^11–17^.

In this survey, we present a proof-of-principle dual-energy phantom radiography experiment, evaluating the diagnostic value of material decomposition images in musculoskeletal diagnosis, which has been enabled by using a new class of hybrid-pixel photon-counting imaging detectors.

Photon-counting detectors (PCDs) were originally developed at CERN for the application as particle tracking detectors in high-energy physics^18,19^. Since the concept provides several advantages over the clinically commonly used energy-integrating flat-panel detector technology, great efforts have been made during the last years to make the technology suitable for use in state-of-the-art imaging applications^20,21^. While flat-panel detectors^22,23^ utilize a scintillator crystal to convert X-rays into visible light which is eventually detected by an array of photodiodes, in PCDs the incident radiation is directly converted into an electrical signal using a semiconductor sensor layer. Thereby, this absence of a separate layer to convert X-rays into light generally results in a higher spatial resolution as the image is less blurred during the signal formation process^24^. Apart from the signal formation process, both detector types strongly differ regarding the processing of the acquired electrical signal. In flat panel based detector systems the electrical charge generated in the photodiodes within one readout cycle is integrated resulting in a loss of spectral information^25^. In contrast, PCDs have pixel-wise readout electronics which allow to process the signal generated by each photon individually. This is realized by comparing the electrical pulse generated by a photon to a threshold and only incrementing the attached counting logic if the registered pulse exceeds the set threshold level^26^. The described implementation provides two advantages. Firstly, setting the threshold higher than the electronic noise level results in a complete elimination of readout noise. Secondly, as the height of the electrical pulse is proportional to the energy deposited by a photon, the implementation of several thresholds allows to sort the incoming photons into distinct energy bins, which provides partly energetically resolved measurements^27^. A comprehensive review on the technical principles and clinical applications of photon counting detectors is given in Refs.^28,29^.

As a sample, we used an ex-vivo human hand. For imaging, a basis material decomposition algorithm^9^ was applied to obtain a bone- and a soft-tissue-equivalence image and a bone-tissue-overlay image.

We performed a reader study in which three experienced radiologists evaluated the diagnostic value and image quality of the two material images (bone and tissue) and of the bone-tissue-overlay image and compared them to the conventional X-ray image of the same sample.

## Results

**Figure 1:**
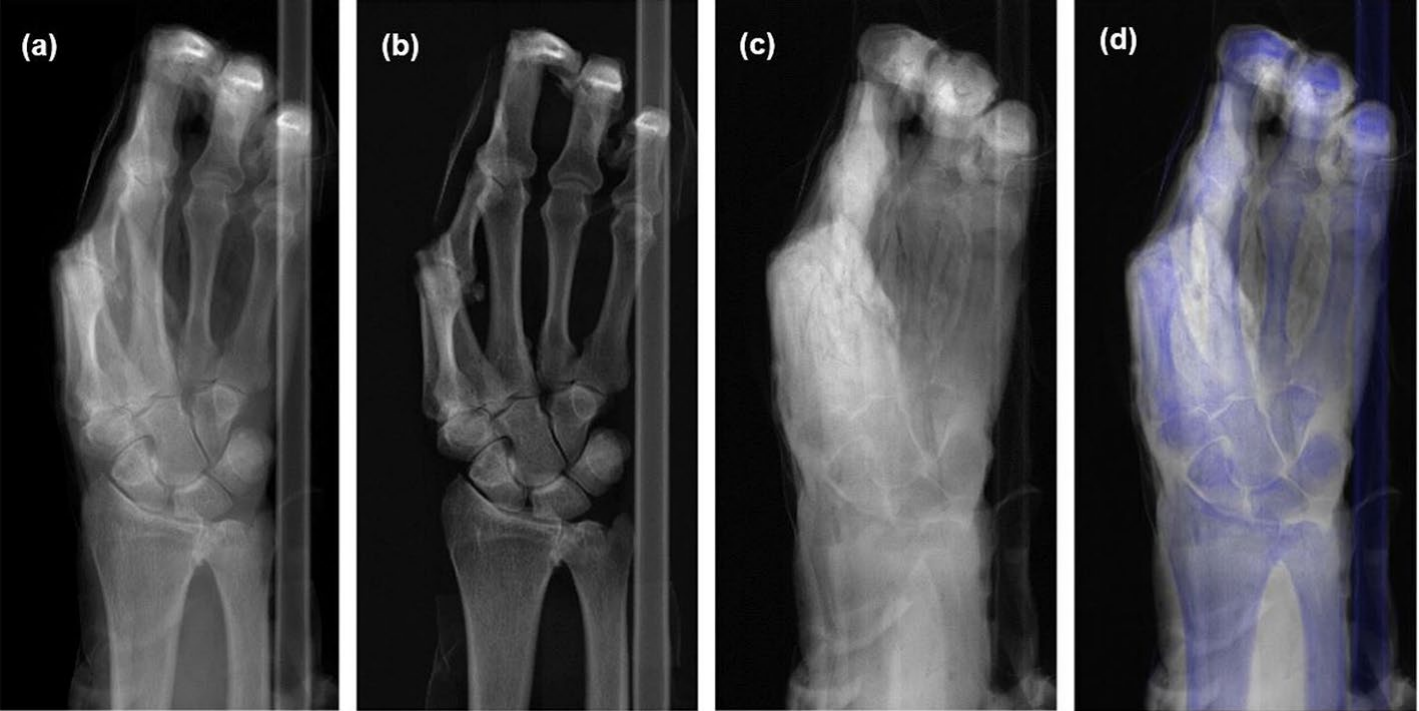
Multiple-contrast X-ray radiographs of an ex-vivo human hand, generated by a spectral photon-counting detector. (**a**) Conventional radiograph image of an ex-vivo human hand. (**b**) Bone- and (**c**) soft-tissue-equivalence image obtained from material decomposition. (**d**) Bone-tissue-overlay image generated by superposing the two basis component images. In the overlay image, the bone-components are coloured in blue.

Figure 1 shows the conventional radiograph, the basis material images and the bone-tissue-overlay image of an ex vivo human hand and the distal parts of radius and ulna. The proximal, intermediate and distal phalanx of the index finger of the hand was ablated post-mortem. The uncommon posture of the hand was caused by the rigor mortis and results in a stacked position of the thumb and the metacarpal bone of the index finger. In addition, the intermediate and distal phalanges of the fingers two to five are flexed and superposed in the projection, resulting in a diminished accuracy and diagnostic value in these areas. The images show long bones (radius, ulna and metacarpals) as well as short bones (all carpal bones) allowing the observers to consider different bone types in their evaluation.

The conventional radiograph is shown in Fig. 1a. Figure 1b,c show the bone and tissue equivalence image, respectively. Figure 1d shows the bone-tissue-overlay image generated by superposing the two basis material images. In the overlay image, the bone-components are coloured in blue.

The three observers evaluated the image quality and diagnostic potential of each image individually as well as the image quality and diagnostic potential of the combination of both basis material images (bone and tissue) and of the combination of all three reconstructed images (basis component images and bone-tissue-overlay image). All three observers also evaluated the image quality and diagnostic potential of the corresponding conventional X-ray image (Table 1).

**Table 1:** Quality criteria and descriptive rating scales used for the reader study.

**Image quality for evaluation of bone structures**				
5 = excellent	4 = good	3 = moderate	2 = bad	1 = not appropriate/applicable
**Visualization of diagnostic details**				
5 = excellent	4 = good	3 = moderate	2 = bad	1 = not appropriate/applicable
**Artefacts**				
5 = no	4 = minor	3 = major	2 = bad	1 = unacceptable
**Overall image quality**				
5 = excellent	4 = good	3 = moderate	2 = bad	1 = unacceptable
**Diagnostic acceptability**				
5 = fully acceptable	4 = probably acceptable	3 = acceptable only under limited conditions	2 = bad	1 = unacceptable

An overview of the complete results of the reader study is shown in Table 2. All three observers evaluated the bone-equivalence image to have a higher image quality than the conventional radiograph for assessing bony structures and a better visualization of diagnostic details, such as trabecular structure or compact bone limits, corresponding to a detail level commonly used in clinical practice at which the differentiability of trabecular and compact bone structure is an important requirement as to the quality of conventional X-ray images in clinical routine^30^. In addition, one of the readers considered the overall image quality of the bone-equivalence image to be better than of the traditional X-ray image while the other two readers did not find an appreciable difference between the two images as to this criterion. Also, all three observers considered the diagnostic acceptability of the bone-equivalence image to be higher than the one of the conventional X-ray image referring to the evaluation of the bony structure.

**Table 2:** Detailed results of the reader study.

	Reader 1	Reader 2	Reader 3	Average score	Standard deviation
**Conventional X-ray (63 points)**					
Image quality for evaluation of bony structure	4	4	4	4.00	0.00
Visualization of diagnostic detail	4	4	4	4.00	0.00
Artefacts	5	5	4	4.67	0.47
Overall image quality	5	4	4	4.33	0.47
Diagnostic acceptability	4	4	4	4.00	0.00
**Bone-equivalence image (73 points)**					
Image quality for evaluation of bony structure	5	5	5	5.00	0.00
Visualization of diagnostic detail	5	5	5	5.00	0.00
Artefacts	5	4	5	4.67	0.47
Overall image quality	5	4	5	4.67	0.47
Diagnostic acceptability	5	5	5	5.00	0.00
**Tissue-equivalence image (30 points)**					
Image quality for evaluation of bony structure	1	1	1	1.00	0.00
Visualization of diagnostic detail	1	1	1	1.00	0.00
Artefacts	4	4	3	3.67	0.47
Overall image quality	2	3	2	2.33	0.47
Diagnostic acceptability	3	2	1	2.00	0.82
**Bone-tissue-overlay image (47 points)**					
Image quality for evaluation of bony structure	3	2	3	2.67	0.47
Visualization of diagnostic detail	3	3	3	3.00	0.00
Artefacts	4	4	3	3.67	0.47
Overall image quality	3	3	2	2.67	0.47
Diagnostic acceptability	4	4	3	3.67	0.47
**Bone-tissue-image (74 points)**					
Image quality for evaluation of bony structure	5	5	5	5.00	0.00
Visualization of diagnostic detail	5	5	5	5.00	0.00
Artefacts	5	4	5	4.67	0.47
Overall image quality	5	5	5	5.00	0.00
Diagnostic acceptability	5	5	5	5.00	0.00
**Bone-tissue-overlay-image (74 points)**					
Image quality for evaluation of bony structure	5	5	5	5.00	0.00
Visualization of diagnostic detail	5	5	5	5.00	0.00
Artefacts	5	4	5	4.67	0.47
Overall image quality	5	5	5	5.00	0.00
Diagnostic acceptability	5	5	5	5.00	0.00

For each image combination subject to evaluation, the table shows the rating of each criteria and each reader, the overall score obtained by summing up all points obtained, and the average score with corresponding standard deviation obtained for each quality criteria subject to analysis.

The tissue equivalence image as a stand-alone image was not considered to be of major diagnostic value. Besides the fact that this image is not appropriate for the evaluation of bony structure nor for the visualization of diagnostic details of the bone, two of the three readers considered the overall image quality to be bad, while one considered it to be moderate. Furthermore, one of the readers considered the diagnostic acceptability to be valid only under limited conditions while the other two readers rated it to be bad or even unacceptable.

The overlay image was also not considered to yield any advantages regarding the diagnostic value and image quality as a stand-alone image. The readers rated the overlay image with a lower score than the bone-equivalence image and even lower than the conventional radiograph image in all five categories.

The additional information provided by the simultaneous viewing of the soft-tissue- and the bone-equivalent image did not relevantly improve the observers' assessment compared to the visualization of the bone-equivalent image alone. Only one of the three observers considered the overall image quality to be better if visualizing both images simultaneously. All other criteria were evaluated equally when only observing the bone-equivalence image alone than when observing both basis material images.

The additional information of the overlay image, when observed simultaneously with the two basis component images did not provide any additional diagnostic value or image quality improvement. All three observers granted the combination of bone image, soft tissue image and overlay image the exact same ratings than the sole combination of the bone and the soft tissue image.

**Figure 2:**
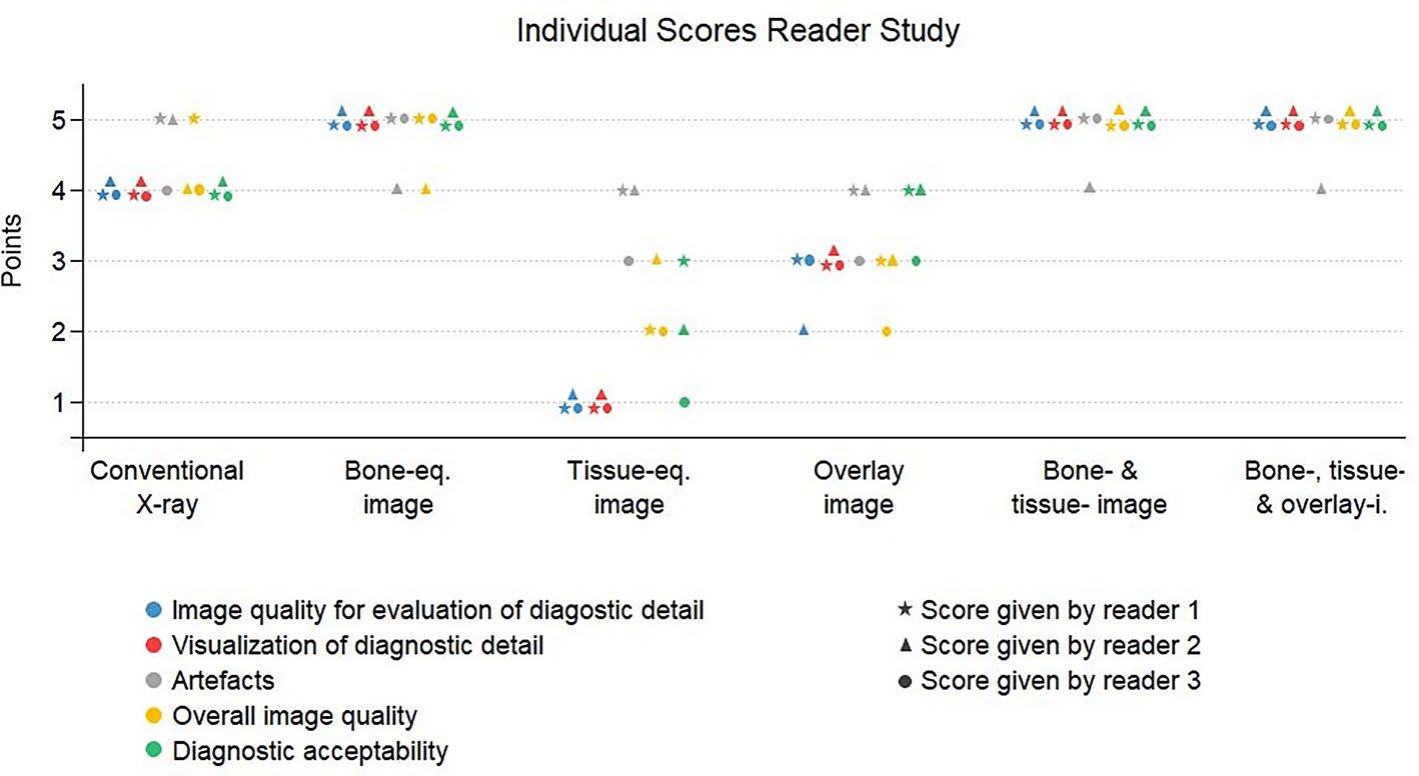
Individual scores reader study. Scatter plot showing the ratings given by each of the 3 readers to each image or image combination for each of the quality criteria subject to evaluation, i.e. image quality for evaluation of bony structure (blue), visualization of diagnostic detail (red), artefacts (grey), overall image quality (yellow) and diagnostic acceptability (green). Best results were obtained by the combination of bone- and tissue-image and by the combination of bone- and tissue- and overlay-image (green), closely followed by the bone-equivalence image alone.

Figure 2 shows a scatterplot indicating the individual ratings assigned by each of the 3 readers to each image or image combination and for each quality criteria subject to this study. The figure illustrates the distribution of the scores assigned by the different readers to each image and quality criteria and it shows that the 2 image combinations (i.e. bone- and tissue-image and bone- and tissue- and overlay-image) obtained the best overall scores, followed by the bone-equivalence image stand-alone, that obtained equal results except for the overall image quality, where its rating is slightly lower.

**Figure 3:**
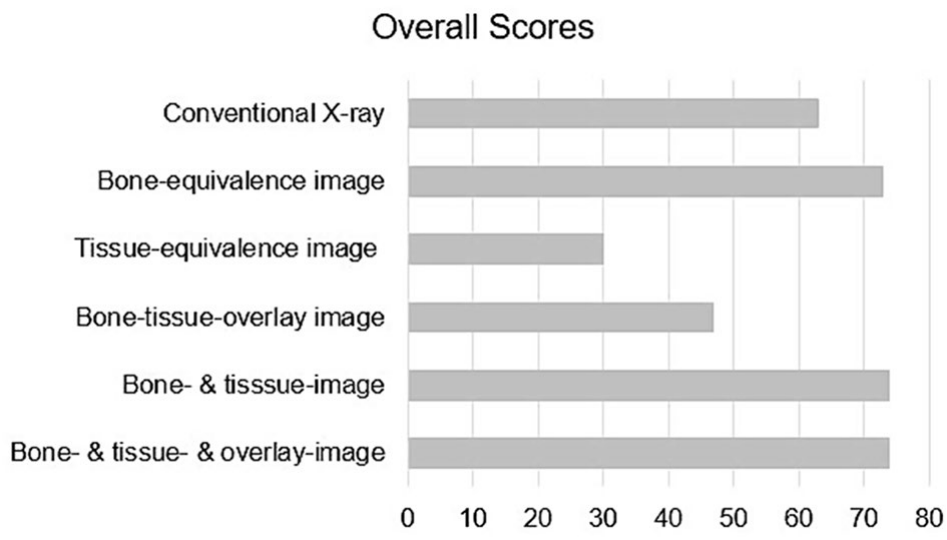
Overall scores reader study. Overall scores obtained by the different images and image combinations subject to evaluation. The bone equivalence image as well as the image combinations show best results yielding an improvement when compared to the conventional X-ray image.

Figure 3 shows the overall scores obtained by the different images or image combinations. The conventional radiograph image obtained a total score of 63 points out of the maximum of 75 possible points in the sum of all quality criteria. The bone-equivalence image obtained 73 of a maximum of 75 possible points in the sum of all quality criteria. Since one of the readers detected a slight improvement in the overall image quality, the combination of bone-equivalence image and tissue equivalence image obtained 74 of a maximum of 75 possible quality criteria points. The addition of the bone-tissue-overlay image did not provide any additional value.

## Discussion

Conventional X-ray images are the state-of-the-art tool used for the diagnosis of fractures and other musculoskeletal lesions in clinical routine. Though small lesions such as microfractures or minor non-displaced fractures are not always easy to be identified in conventional X-ray images, particularly at the diagnosis of hand and wrist fractures, e.g. the scaphoid, which may lead to insufficient treatment and rehabilitation^1–4^.

Material decomposition methods yield the possibility to filter the conventional X-ray images in order to obtain a bone-equivalence image showing only the attenuation caused by bony-material and excluding the effects caused by soft tissue, which are overlaying in conventional radiography^9^, promising a potentially higher image quality and an increased level of diagnostic confidence and hence a prospectively improved approach to the limitations faced in the musculoskeletal diagnostics with conventional radiographies.

Dual-energy radiography has been analysed regarding the advantages of tissue-equivalence images in subtraction chest radiography^11–17^ and R. Panta et al. have presented first studies concerning the diagnostic quality of spectral CT Images of musculoskeletal body parts^31^. In our study, we present first results of bone equivalence radiographs offering improved image quality for the diagnostic evaluation of bony structures.

Our study shows that basis material decomposition and particularly bone-equivalence images have a high potential of improving the diagnostic outcome of radiograph examinations of musculoskeletal disorders, suggesting considerably better image quality and diagnostic detail of bony structures and a higher overall image quality and diagnostic acceptability than conventional radiography.

While these results are very promising, the limitations and drawbacks of the study must be considered.

Firstly, our study was based on only one sample of an ex-vivo human hand. Further samples of the same and other bony structures of the human body should be examined for revalidation of the obtained results and for the evaluation of the transferability of these results to other anatomical structures such as a leg or an arm, where the amount of soft tissue is considerably higher compared to the hand.

Additionally, the used ex-vivo human hand did not contain any fractures, microdamage or tumours, as to the diagnostic findings of a laboratory CT-scan of the hand by two experienced radiologists. Although the image quality and diagnostic detail of the bony structure has been assessed to be better in bone-equivalence images than in conventional radiography, future studies will be necessary to evaluate the clinical potential of bone-equivalence radiographs for the detection of osseous pathologies such as e.g. fractures, tumours and infectious diseases.

Furthermore, the unnatural position of the hand caused by rigor-mortis resulting in a superposition of the projections of some bony structures, as well as the ablation of the index finger is unfavourable to the image analysis. Yet the image includes enough long and short bones visible without restrictions in order to evaluate the image quality in an adequate manner. Regardless, an evaluation of a human hand sample in an adequate position should be conducted in order to extend the evaluation of the bony structures and include the small long bones of the distal phalanxes of the fingers.

Finally, it has to be mentioned, that the reader study was conducted by a small number of observers. Though the ratings of the three readers show a clearly equal tendency and an obvious result, the significance of the study ought to be enhanced by a larger pool of observers in further surveys.

In conclusion, our results show that spectral photon-counting material decomposition and the resulting bone- and tissue-equivalence images provide promising improvements in the image quality and diagnostic value of musculoskeletal radiograph images.

## Methods

The ex-vivo human hand was retrieved from a human body donor who had given written informed consent to donate his body after deceasing for medical research and medical education after his death in accordance to German law and international ethical guidelines. All experimental protocols were approved by the ethics committee at Klinikum rechts der Isar (application number 455/18 S). The sample was preserved in formalin.

**Figure 4:**
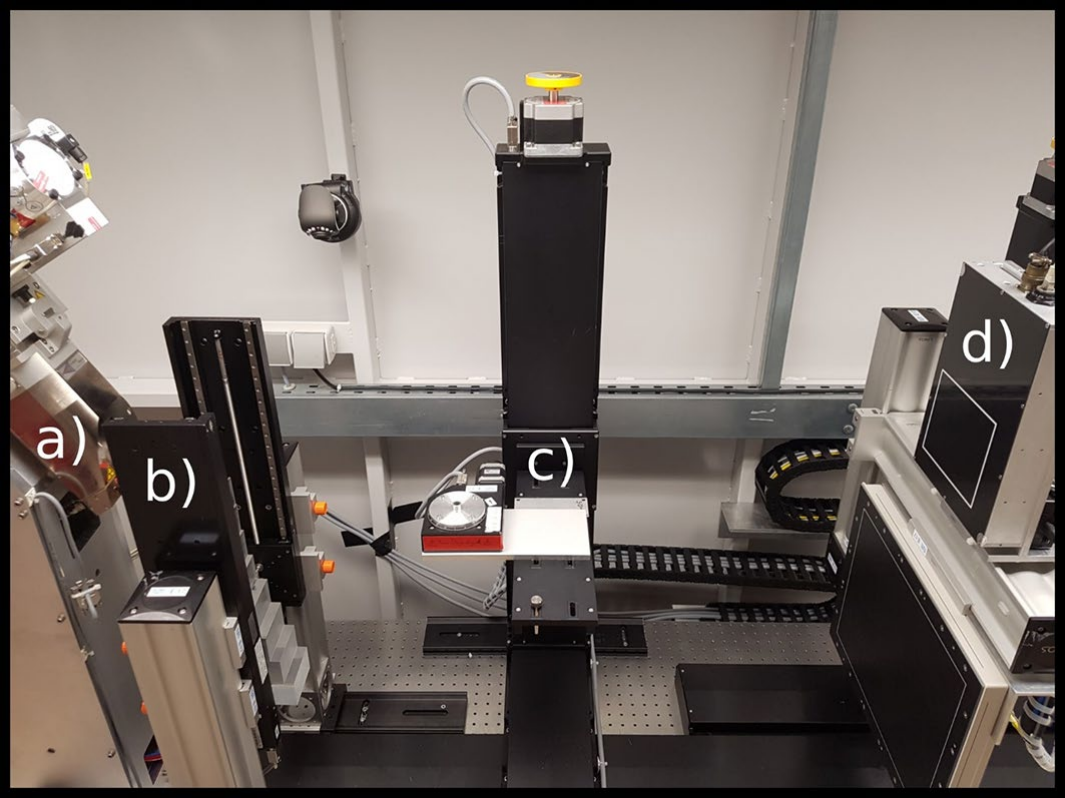
Experimental set-up. (**a**) X-ray source. (**b**) Linear-stages with calibration phantoms. (**c**) Sample stage. (**d**) Photon-counting detector.

The experimental measurements were performed at a stationary, lab-bench CT setup, shown in Fig. 4. The X-ray source (XWT-160 SE, X-RAY WorX, Garbsen, Germany) has a tungsten target and a 2 mm thick beryllium window. The image data was acquired with a commercially available photon-counting detector (Flite X1, Direct Conversion, Danderyd, Sweden)^32^, with two thresholds per pixel and an integrated charge-sharing correction, which allows for dual-energy acquisition in a single shot. The detector has a cadmium telluride (CdTe) sensor with a thickness of 750 µm providing a high quantum efficiency in the relevant energy range. Due to the integrated charge-sharing correction^33^ the detector has a box-like point-spread-function^34^ resulting in an effective resolution of 100 µm and a field-of-view of 15.3 × 1.3 cm^2^. Based on the limited field-of-view of the detector the sample was scanned in vertical direction. The acquisition parameters are listed in Table 3. The position of the energy thresholds was selected with the objective of obtaining minimum noise in the decomposed basis-material images. Thereby, the optimal settings depend on the source spectrum and the composition and size of the measured sample. The estimation of the optimal threshold setting was done based on an approach described in Ref.^35^.

**Table 3:** Acquisition parameters of the photon counting detector used for the experimental measurements of this study.

Tube voltage	110 kVp
Tube loading	3.5 mAs
Beam filtration	0.1 mm Cu
Threshold position	23, 55 keV
Source to isocentre	134 cm
Isocentre to detector	16 cm
Physical pixel size	100 µm

The acquired dual-energy data was decomposed into photoelectric absorption and Compton scatter equivalent line-integrals using a maximum-likelihood method^36^. Afterwards, those line-integrals were transformed to bone and soft-tissue equivalent line-integrals by a change of the corresponding vector basis^10^. The maximum-likelihood estimation of the basis-material line-integrals was done with an empirical forward-model^9^. Prior to the acquisition of the sample the free parameters of the utilized forward-model were tuned by calibration measurements as described in reference^9^. Thereby, step wedges made of polyvinylchloride (PVC) and polyoxymethylene (PMMA) were used to create a set of 64 calibration points. The step wedges were mounted to movable linear stages to acquire dual-energy data of the 64 thickness combinations of 0–32 mm PVC and 0–64 mm PMMA. The materials PVC and PMMA were chosen for calibration since their energy-dependent attenuation properties resemble those of bone and soft-tissue accurately. The quantitative accuracy of the decomposed line-integral values was evaluated in prior studies in numerical simulations^8^ as well as experimental measurements^37^.

Three experienced radiologists with 4, 5 and 6 years of experience in musculoskeletal diagnosis, respectively, assessed the image quality and diagnostic value of the conventional X-ray image, of the two material images (bone and tissue) and of the bone-tissue-overlay image. Furthermore, the observers also evaluated the diagnostic value of the combination of both material images as well as of the combination of all three images (i.e. bone, soft tissue and bone-tissue-overlay).

The readers assessed the images by rating the following quality criteria:

- Image quality for evaluation of bony structures (IQBS)
- Visualization of diagnostic details (VDD) such as trabecular structure and compact bone limits
- Artefacts (A)
- Overall image quality (OIQ)
- Overall diagnostic acceptability (DA)

The trabecular structure and compact bone limits were analysed according to the detail level corresponding to the one commonly used in clinical practice for the diagnosis of conventional radiography images and at the standard at which the differentiability of trabecular and compact bone structure is an important requirement to the quality of conventional X-ray images in clinical routine^30^. It does not pretend to meet the resolution and detail level of existing technologies for the detailed evaluation of trabecular structures for the evaluation of e.g. osteoporosis by techniques like fractal analysis^38^.

The evaluation was conducted for each image separately with a delay time of 2 weeks and in randomized order to minimize recall bias. In the first 4 sequences only one of the images (conventional radiograph, bone-equivalence image, tissue-equivalence image and bone-tissue-overlay image, respectively) was evaluated. In the fifth sequence bone- and tissue-equivalence image were available to the readers and evaluated conjointly and in the last sequence bone-, tissue- and overlay-image were available to the readers and evaluated conjointly. The evaluation was done by using a 5-point descriptive rating scale for each quality criteria, ranging from 1 point (poor quality) to 5 points (excellent quality). The descriptive ratings for the five above named evaluation criteria are listed in detail in Table 1.

The results of the reader study were interpreted by considering the results for each quality criteria separately (Fig. 2) as well as by observing the obtained overall scores of each image or image combination (Fig. 3). The scatterplot in Fig. 2 shows all individual scores assigned to the images and quality criteria by the readers. In order to distinguish between the ratings given by the different readers to each image or image combination, each reader has been assigned a different shape indicator in the scatter plot (star for reader 1, triangle for reader 2 and circle for reader 3). The colours of the shapes of the scatter plot indicate the quality criteria that was rated. The overall scores shown in Fig. 3 were obtained by summing up the total number of points received by each image or image combination subject to evaluation. For each image or image combination all points obtained by all readers and for any of the quality criteria were added to obtain the overall score attributed to the image or image combination.
